# Evaluating a novel vertical traction device for early closure in open abdomen management: a consecutive case series

**DOI:** 10.3389/fsurg.2024.1449702

**Published:** 2024-08-13

**Authors:** J. Dohmen, D. Weissinger, A. S. T. Peter, A. Theodorou, J. C. Kalff, B. Stoffels, P. Lingohr, M. von Websky

**Affiliations:** ^1^ Department of Surgery, University of Bonn, Bonn, Germany; ^2^ Department of Surgery, Ippokrateio University Hospital Athens, Athens, Greece; ^3^ Department of General and Visceral Surgery, Trauma Surgery, Cellitinnen-Hospital Holy Spirit, Cologne, Germany; ^4^ Department of General, Visceral and Transplantation Surgery, RWTH Aachen University Hospital, Aachen, Germany

**Keywords:** open abdomen (OA), abdominal compartment syndrome (ACS), temporary abdominal closure (TAC), definitive fascial closure (DFC), vertical traction device (VTD), negative pressure wound therapy (NPWT)

## Abstract

**Background:**

In emergency surgery, managing abdominal sepsis and critically ill patients with imminent abdominal compartment syndrome (ACS) using an open abdomen (OA) approach has become standard practice for damage control. To prevent significant complications associated with OA therapy, such as abdominal infections, entero-atmospheric fistula (EAF), and abdominal wall hernia formation, early definitive fascial closure (DFC) is crucial. This study aims to assess the feasibility of a novel device designed to facilitate early fascial closure in patients with an open abdomen.

**Methods:**

Between 2019 and 2020, nine patients undergoing open abdomen management were enrolled in this study. All patients were treated using vertical mesh-mediated fascial traction combined with a novel vertical traction device (VTD). Data from these cases were collected and retrospectively analyzed.

**Results:**

In this study, all patients were treated with OA due to impending ACS. Three patients died before achieving DFC, while the remaining six patients successfully underwent DFC. The mean number of surgical procedures after OA was 3 ± 1, and the mean time to DFC was 9 ± 3 days. The use of the VTD in combination with negative pressure wound therapy (NPWT) resulted in a 76% reduction in fascia-to-fascia distance until DFC was achieved. The application of the VTD did not affect ventilation parameters or the Simplified Acute Physiology Score II (SAPS II), but intra-abdominal pressure (IAP) was reduced from 31 ± 8 mmHg prior to OA to 8.5 ± 2 mmHg after applying the device. The primary complication associated with the device was skin irritation, with three patients developing skin blisters as the most severe manifestation.

**Conclusion:**

Overall, the novel VTD appears to be a safe and feasible option for managing OA cases. It may reduce complications associated with OA by promoting early definitive fascial closure.

## 1 Introduction

In recent years, damage control procedures have been increasingly applied in emergency and intensive care settings with critically ill patients (1). The open abdomen (OA) approach is a damage control surgery involving an incision and intentional laparostomy to facilitate decompressionand prevent abdominal compartment syndrome (ACS) (2). Primary indications for OA include abdominal sepsis, trauma involving the abdomen, mesenteric ischemia, acute severe pancreatitis (1). Notably, in trauma cases alone, approximately 25% of emergency laparotomies are not eligible for primary fascial closure (3). ACS is characterized by sustained intra-abdominal pressure exceeding 20 mmHg, accompanied by new organ dysfunction or failure (4). In critically ill patients the incidence of ACS is 3% while extracorporeal membrane oxygenation (ECMO) treatment raises this risk to about 10% due to fluid shifts and visceral edema, leading to intra-abdominal hypertension (IAH) and consecutively ACS (5, 6). Since ACS can result in multi-organ failure (MOF), severe ventilation issues and complications related to ECMO therapy, immediate surgical decompression is essential (7, 8). Despite this, the open abdomen remains a last resort option due to its non-anatomical nature and associated mortality rates of 12%–40% (9). Furthermore, OA is linked with numerous major complications, including MOF in up to 30% of cases, intra-abdominal abscesses or surgical site infections in 20%–83%, entero-atmospheric fistula (EAF) formation in 10%–20% and ventral abdominal hernia in 20%–30% of patients (3, 10–13). The incidence of these complications varies based on the indication for OA (e.g., trauma vs. non-trauma) and the disease course. Generally, a delay in fascial closure correlates with a higher rate of complications (1, 14). Given the imperative to achieve early fascial closure, risk factors for failed abdominal closure include sepsis, EAF, intra-abdominal abscess, prolonged OA management and the number of surgical procedures (11, 15–17). During OA, temporary abdominal closure (TAC) is employed to protect the abdominal viscera (18). Over the past decades various TAC techniques have been used. According to the guidelines of the World Society for Emergency Surgery (WSES) (1) vacuum-assisted wound closure and (horizontal) mesh mediated facial traction (VAWCM) is currently the suggested method, achieving definitive fascial closure (DFC) rates of over 80% (12, 19, 20). However, even with negative pressure wound therapy (NPWT) and VAWCM as the current standards, there remains a high incidence of incisional hernias, ranging from 20% to 40% (21, 22). Additionally, NPWT alone is limited in its ability to medialize fascial margins. Hence, new concepts to prevent fascial retraction, facilitate early definitive fascial closure and reduce the incidence of incisional hernia are necessary (23).

In traditional horizontal VAWCM, the volume of the abdominal cavity is reduced as the fascial margins are approximated. When an increase in cavity volume is required, the tension of the mesh- mediated traction must be relaxed, which can compromise the effectiveness of fascial closure. In contrast, vertical traction offers a more dynamic approach by preserving the volume of the abdominal cavity while simultaneously extending the fascial margins. This method theoretically optimizes the conditions for achieving early and effective fascial closure. A new vertical traction device (VTD) that employs vertical mesh-mediated traction on the fascial margins has recently been shown to decrease fascial tension and facilitate abdominal closure (24–27). This study aimed to evaluate the feasibility and safety of the novel VTD in our cohort and determine if it promotes early fascial closure in patients with OA.

## 2 Material and methods

### 2.1 Device and application

The VTD (fasciotens®Abdomen, Fasciotens GmbH, Essen, Germany) primarily applies dynamic vertical directed tractive force along both facial margins via an external support system. The system comprises a scaffolding, a suture retention frame for suture clamping and two cushioned support bases that evenly distribute weight over the thorax and the anterior pelvic ring.

**Figure 1:**
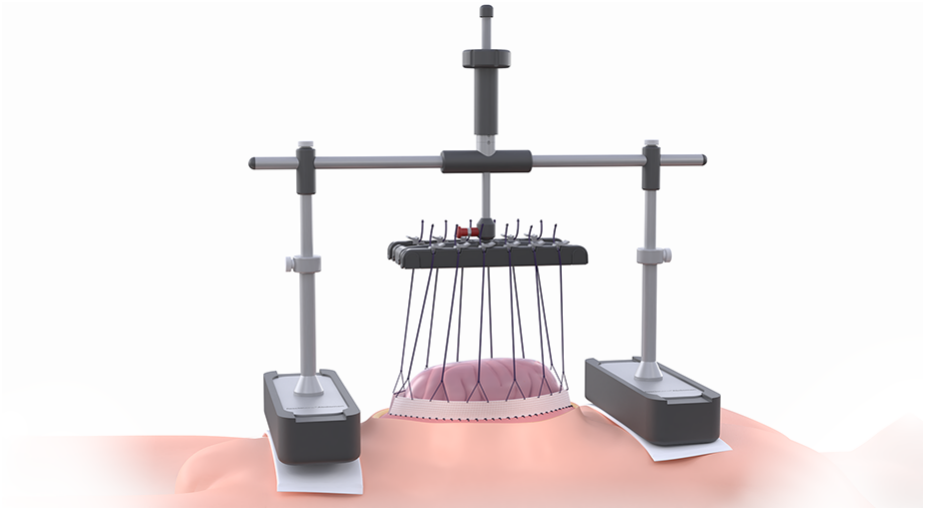
fasciotens®Abdomen device (with kind permission of Fasciotens GmbH, Essen, Germany).

Once the abdomen is accessed through a transverse or longitudinal laparotomy, a vicryl mesh is sutured to both fascial margins and incised longitudinally. Six looped sutures are then placed along each mesh margin to evenly distribute traction forces along the entire length of the fascial margins. The looped sutures are locked on each side via clamping. The suture retention frame is then attached to the longitudinal beam of the scaffolding, which rests on cushioned support bases along the thorax and the anterior pelvic ring. The height and length of the scaffolding can be adjusted to facilitate attachment. Fascial traction forces can be modified using a screw-like mechanism, up to approximately 100 Newton according to the manufacturer’s specifications. This setup pulls the fascial margins vertically to counteract natural muscle retraction forces. Additionally, the suspension of the fascial margins decreases intra-abdominal pressure, allowing for the application of vacuum-assisted TAC between the tent-shaped suture suspensions. In case of an emergency, a red locking bolt can be pulled to release the suture retention frame, allowing the scaffolding and bases to be removed immediately (Figure 1).

### 2.2 Device application algorithm

Once patients developed ACS, they underwent emergency decompression laparotomy with initial application of either polyurethane foil (Medline International Germany GmbH, Germany) or ABThera^TM^ (3M Company, St. Paul, MN, USA) for TAC. During the second look surgery, each patient was evaluated for the application of the VTD. If further OA therapy was required, the VTD was implemented as described above. Subsequent surgical revisions were conducted as needed based on the abdominal situation. During each surgery maximal tractive forces (approximately 100 Newton) were applied over 30 min with full muscle relaxation and patients were evaluated for DFC. Visceral protection was ensured by using polymeric membrane dressings (PolyMem® Wic® Cavity Filler, Ferris Mfg. Corp., Fort Worth, TX, USA) or polyurethane foil as protective layer. The only intraoperative reason to halt VTD therapy was the presence of complex adhesions of viscera to the abdominal wall.

After transfer to the ICU, tractive forces (approximately 40–50 Newton) were applied in cyclical fashion for 5 h with 1-hour relief intervals, totaling in 20 h of tractive forces within a 24-hour period. During relief intervals, the VTD was removed, and the sutures were cross-fixed in the suture holder to maintain some tension and prevent fascial retraction.

### 2.3 Technical analysis

For Statistical analysis Statistical Package for Social Sciences software (SPSS®, Vers. 17.0, Chicago, Il, USA) and Excel (Microsoft®, Version 16.44, Redmond, WA, USA) were used. Descriptive statistical tools were applied to the data. The mean was used as a measure of central tendency and the range and standard deviation were used as measures of dispersion. The large language model ChatGPT 4 (OpenAI, San Francisco, USA, RRID:SCR_023775) was used for language revision.

## 3 Results

### 3.1 Patients characteristics

During a two-year period, nine critically ill patients undergoing OA management were enrolled in this study. The general characteristics of the patients are presented in Table 1. The cohort consisted of five males (56%) and four females (44%), with a mean age of 52 ± 20 years, ranging from 23 to 78 years (Table 1). Despite the age diversity, all patients received OA treatment to prevent ACS. The etiologies of IAH included acute respiratory distress syndrome (ARDS) with MOF, ECMO therapy, peritonitis, bowel ischemia and postoperative bleeding with hematoma (Table 1). Six patients (66%) were on dialysis, eight (89%) required vasopressor therapy and invasive mechanical ventilation, and all patients received antibiotics prior to OA treatment. The average ASA score of the study population exceeded 3, indicating high morbidity and surgical risk (Table 1) (28). This elevated ASA score was primary due to acute illness rather than chronic disease, as common chronic diseases like diabetes mellitus, myocardial disease, arterial hypertension, chronic obstructive pulmonary disease (COPD) and chronic renal insufficiency were present in only 11% to 44% of patients (Table 1).

**Table 1:** Patient characteristics and Co-morbidities.

Total	*n* = 9	
Gender		
Male	5	56%
Female	4	44%
Age (years)	52 ± 20	
BMI (kg/m2)	26 ± 4	
Reason for OA		
Imminent ACS	9	100%
Etiology		
ARDS	2	22%
ARDS + ECMO	3	33%
Peritonitis	2	22%
Bowel ischemia	1	11%
Bleeding	1	11%
ASA		
III	3	33%
IV	5	56%
V	1	11%
Previous Abd. Surgery	6	67%
Comorbidities		
Diabetes mellitus	1	11%
Myocardial disease	4	44%
Art. Hypertension	3	33%
COPD	2	22%
Renal insufficiency	3	33%
ICU Treatment (prior to first surgery)		
Dialysis	6	67%
Ventilation	8	89%
ECMO	3	33%
Antibiotics	9	100%
Vasopressors	8	89%

BMI, body mass index; OA, open abdomen; ACS, abdominal compartment syndrome; ARDS, acute respiratory distress syndrome; ECMO, extracorporeal membrane oxygenation; ASA, American society of anesthesiologists; COPD, chronic obstructive pulmonary disease; ICU, intensive care unit.

### 3.2 Early fascial closure with VTD

The VTD was successfully applied in all nine cases, however three patients died before DFC could be achieved (Table 2). Causes of death included myocardial infarction, cardiac arrest and ARDS with hypoxia. There was no sign of being associated with the use of VTD. Alle patients were intubated during the traction process. DFC was successfully achieved in the remaining six patients (Table 2) without component separation or mesh reinforcement. Early fascial closure is essential to minimize the need for multiple surgical procedures and reduce complications associated with OA, which escalate with time and the number of revisions (14). In this study, fascial traction with the VTD was applied on average within four days after decompression laparotomy. In five patients, VTD application was initiated within three days post-laparotomy. The mean number of surgical procedures required to achieve DFC with VTD treatment was 3 ± 1, ranging from one to five procedures. DFC was achieved after an average of 9 ± 3 days, ranging from 5 to 14 days (Table 2).

**Table 2:** Open abdomen course.

Total	*n* = 9	
Death prior to DFC	3	33%
DFC	6	67%
Total	*n* = 6	
Number of Revisions	3 ± 1	
Days until DFC	9 ± 3	
FD at OA (cm)	12 ± 2	
FD at DFC (cm)	2.5 ± 3	
Abs. FDR (cm)	10 ± 5	
Rel. FDR (%)	76 ± 31	
NPWT	8	89%

DFC, definitive fascial closure; FD, fascial distance; FDR, fascial distance reduction; NPWT, negative pressure wound therapy.

**Figure 2:**
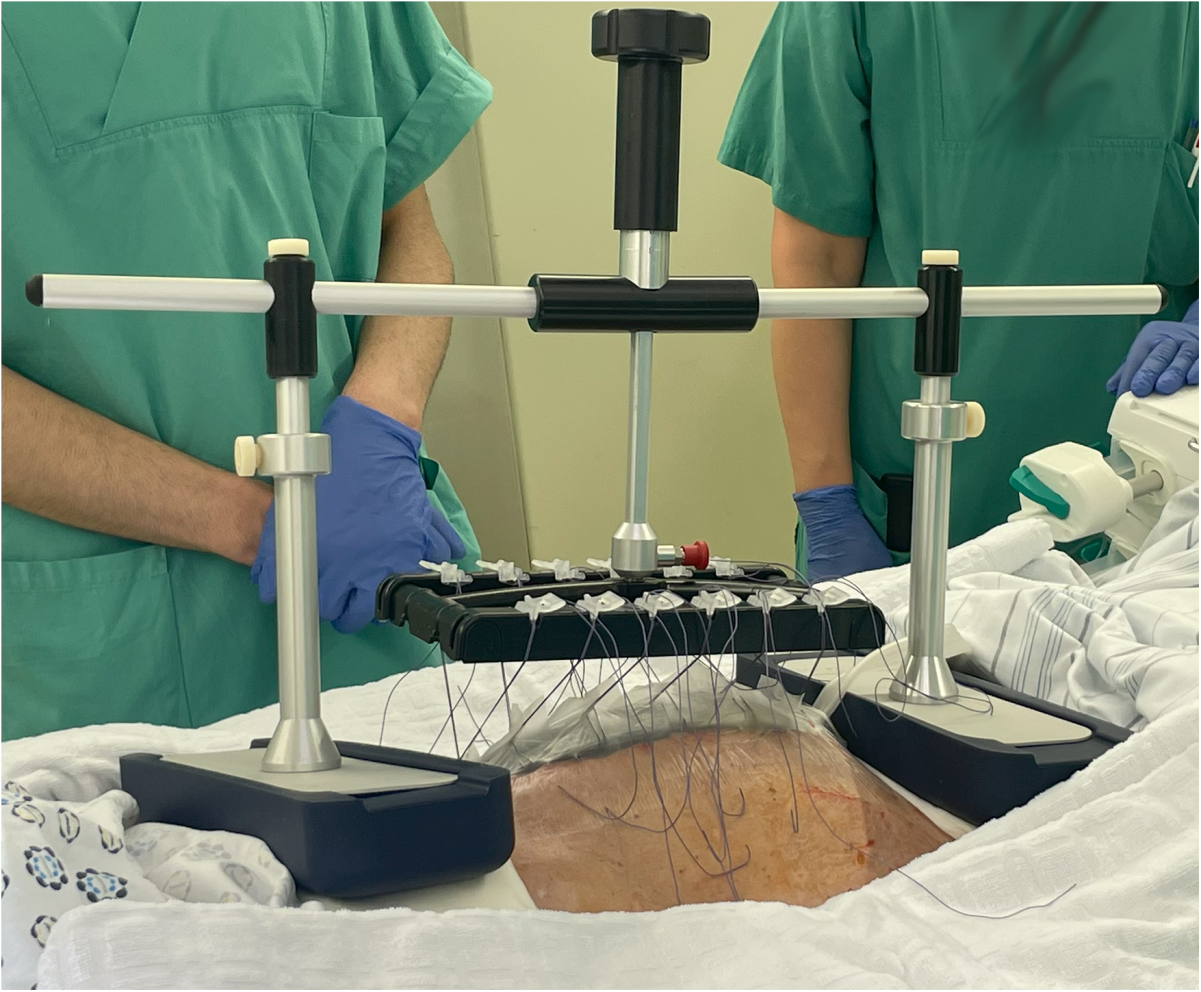
fasciotens®Abdomen device placed on a patient in the intensive care unit (with kind permission of Fasciotens GmbH, Essen, Germany).

To assess the effectiveness of the VTD in preventing fascial retraction and promoting fascial elongation, the fascial distance was measured before and after VTD application. The mean fascial distance decreased from 12 ± 3 cm before VTD application to 2.5 ± 3 cm upon DFC, resulting in an absolute reduction of 10 ± 5 cm (Table 2). Thus, the VTD facilitated a fascial distance reduction of approximately 76% ± 31% (Table 2). TAC during VTD cycles was combined with common NPWT in eight patients without major complications (Figure 2).

### 3.3 Impact on patient physiology and ICU therapy

**Figure 3:**
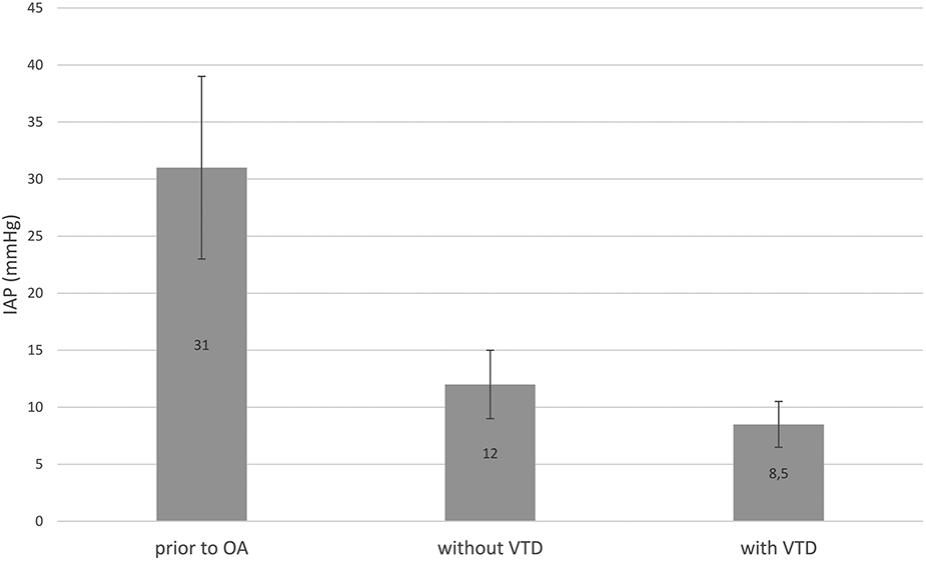
Intra-abdominal pressure (IAP) was measured in mmHg at various timepoints: Prior to laparotomy for ACS, after pressure relief without the VTD and after pressure relief with VTD application. The results are presented as mean values with standard deviations.

To ensure the VTD's feasibility, it was crucial to confirm that the device was safe and effective without impairing patient physiology or intensive care unit (ICU) therapy. The influence of the VTD on IAP was analyzed in five patients with available data. Prior to OA, the mean IAP was 31 ± 8 mmHg. Post-surgical intervention, IAP decreased to 12 ± 3 mmHg without the device and further to 8.5 ± 2 mmHg with the VTD in place (Figure 3).

Additionally, ICU-specific parameters were monitored before and on the second day of VTD application. We did not observe changes in the number of patients on dialysis or invasive mechanical ventilation. The SAPS II Score showed no significant changes with or without the device (Table 3).

**Table 3:** Pathophysiological parameters.

	Prior to OA		With VTD	
SAPS II Score	52 ± 15		50 ± 17	
Dialysis	6	67%	6	67%
Ventilation	8	89%	9	100%

SAPS II, simplified acute physiology score II.

### 3.4 Complications related to device application

Despite the overall safety and feasibility of the VTD, some complications were noted. The major complication in three patients was the development of local skin irritations under the vacuum seal, including blisters in the areas of support base application (Table 4). We therefore established a cyclical application scheme with an intermittent relief of the device for one hour every five hours. No other application-specific complications were reported and there were no occurrences of EAFs, intra-abdominal abscesses or wound infections during VTD use. After fascial closure, no patient experienced secondary wound dehiscence. During follow-up, one patient underwent another laparotomy due to an ischemic colon, resulting in multiple revisions and development of an EAF. Another patient developed secondary subcutaneous wound infection post-DFC. After fascial closure NPWT was maintained until skin closure. Long-term follow-up data on hernia formation was only available for one patient, who did not develop a hernia to date. Fascial dehiscence was not recorded during the hospital stay for all patients.

**Table 4:** Complications.

Total	*n* = 9	
Skin	3	33%
EAF	0	
Abscess	0	
SSI	0	
SFD	0	
Fascial dehiscence	0	

Skin, skin irritations; EAF, entero-atmospheric fistula; Abscess, intra-abdominal abscess formation; SSI, surgical site infection; SFD, secondary fascial dehiscence; Hernia, hernia formation.

## 4 Discussion

As depicted in this study, abdominal hypertension and imminent ACS occurs in a heterogenous patient population with varying etiologies, making it challenging to recruit a homogeneous patient cohort for optimal comparability. Additionally, the patient's physiology and intraabdominal findings dictate the course of OA treatment, rendering standardization nearly impossible. For instance, the possibility for DFC depends on the specific etiology necessitating OA treatment (19). Furthermore, OA management requires a multidisciplinary approach involving teamwork and dedication. Given that OA surgery is often emergent, it frequently involves different surgeons who may not be familiar with the study specifics. Additionally, the ICU team must be educated in device handling, IAP measurement and documentation.

The diversity of parameters measured and the involvement of multiple parties make data collection cumbersome. This is reflected in recent cumulative analysis of OA treatment, which shows adherence to a standardized treatment protocol in only 30% of cases (19). For future studies, it is crucial to implement and distribute a standardized protocol and adequately educate all involved parties.

The primary aim of this study was to evaluate the feasibility of the novel fasciotens®Abdomen VTD in OA treatment. Since abdominal compartment syndrome is a symptom of systemic disease in critically ill patients, it is essential to demonstrate not only the efficacy and handling of the device but also patient safety. Our study showed that despite the critical condition of the patients, the device could be used without major application-specific complications. The main complications observed were local skin irritations. While skin irritations can occur under vacuum seal in critically ill patients (29), the support base of the device can potentially increase skin irritations. Therefore, we followed a cyclical application of the device for five hours with intermittent relief for 60 min. Additionally, cushioning with compresses or similar soft materials can further reduce the risk for skin irritations, as described by the manufacturer. Nevertheless, the device was not always accepted in the ICU due to its initially challenging appearance and handling requirements. Consequently, it is imperative to implement a comprehensive onboarding and training program for ICU staff, including both nurses and physicians, to facilitate the adoption and proficient use of the VTD. Moreover, the VTD did not interfere with ICU treatments or exacerbate patient physiology, indicating its safety even in critically ill patients.

In addition to analyzing the safety and feasibility of the device, a major aim of this study was to evaluate the effectiveness in achieving early fascial closure in OA treatment. According to the European Hernia Society open abdomen registry, the DFC rate with common TAC methods is 71% in per protocol analysis and 57.5% in intention-to-treat analysis. In our study, a 100% DFC rate was achieved in all patients who survived until DFC, surpassing the reported DFC rate of approximately 83%–86% with visceral protective layer (VPL) + NPWT + dynamic closure technique (DCT) therapy (19, 22). Additionally, the mean duration of OA treatment in the multi-center analysis by Willms et al. was 16 ± 24.9 days with an average of 3.9 ± 3.7 surgical procedures, while our data show reduction in both.

Recent years have seen various attempts to improve early DFC in OA. In a study of 165 patients on prognostic factors for OA treatment the DFC rate was 82% with a mean time to DFC of 11 days and an average of five surgical procedures (30). One approach uses an abdominal re-approximation anchor (ABRA®, Access Pro Medical Inc., Augusta, GA, USA) system that employs medial fascial traction. Wang et al. reported 100% DFC rate with an average of two surgical procedures using the ABRA system in trauma patients (31). Another study on the ABRA System reported an 88% DFC rate with an average treatment time of 15 days and a 66% complication rate due to pressure sores (32). Petersson et al. used a vacuum-assisted wound closure and permanent onlay mesh (VAWCPOM) technique, achieving a 100% DFC rate but requiring permanent alloplastic material. The median OA duration was 10 days with three required dressing changes (33).

The VTD used in this study is unique as it applies force vertically. An experimental study in pigs showed a significant decrease in abdominal tension without affecting vitals parameters and ventilation pressure, warranting further evaluation in humans (25). A case report demonstrated successful DFC without significant complications, even in an awake, conscious and spontaneously breathing patient (24). Fung et al. observed a significant decrease in fascia-to-fascia distance of 5 cm within 48 h following device application, with a median time to DFC of seven days and low complication incidence, although their study required twice the number of revisions on average (26). Mones et al. reported a mean time to DFC of 6 days after VTD treatment and a comparable FD at OA (27). We applied traction forces between approx. 40–50 N, whereas both other studies propose traction forces of around 70 N (26, 27). Potentially, the time to DFC is influenced by higher traction forces. Notably, both studies did not include IAP measurements.

In our study, combining the VTD with NPWT was successful in reducing the need for dressing changes due to ascites and wound secretion in eight patients. Given that NWPT increases the risk of EAF, visceral protection was ensured for all patients (34). The anteriorly mediated vector of force simultaneously enabled fascial traction and intra-abdominal pressure relief, particularly beneficial in ECMO therapy and ACS. From an economic standpoint, reducing length of ICU and hospital stay, as well as number of surgeries, is crucial (35). The VTD used in this study has the potential to decrease ICU stay and the number of days and surgical procedures until DFC compared to NPWT, thereby reducing risk of complications associated with surgical interventions. Additionally, early DFC decreases mortality and complications from OA, such as EAF, which has a mortality rate of up to 10%–40% (36, 37).

This small case series was a pilot study and can thus only depict trends. We did not perform a follow up on patients after DFC, so long-term data is only known for one of our patients, who has not developed a hernia to date. Nevertheless, the promising data encourage further studies to evaluate the efficacy of this VTD application compared to the current standard of horizontal mesh mediated NPWT.

In conclusion, fasciotens®Abdomen stands out as the sole device capable of generating vertical dynamic traction. Our findings suggest that although challenging to implement, it may represent a safe and efficient solution for treating OA, potentially mitigating complications linked to OA through the facilitation of early definitive fascial closure. Moreover, it appears that patient physiology, including intra-abdominal pressure and ventilation, remains unaffected by its use.

## Data Availability

The original contributions presented in the study are included in the article/Supplementary Material, further inquiries can be directed to the corresponding author.
